# Heavy Metal and Petroleum Hydrocarbon Contaminants Promote Resistance and Biofilm Formation in *Vibrio* Species from Shellfish

**DOI:** 10.3390/microorganisms13112522

**Published:** 2025-11-02

**Authors:** Gongshi Lin, Yingpeng Li, Ying Qiao, Theerakamol Pengsakul, Guobin Chen, Lixing Huang

**Affiliations:** 1 Key Laboratory of Healthy Mariculture for the East China Sea, Ministry of Agriculture, Fisheries College, Jimei University, Yindou Street 43, Xiamen 361021, China; 2 Xiamen Marine & Fisheries Research Institute, Xiamen 361013, China; 3 Fourth Institute of Oceanography, Ministry of Natural Resources, No. 26, New Century Avenue, Beihai 536000, China; 4 Health and Environmental Research Center (HERC), Faculty of Environmental Management, Prince of Songkla University, Hat Yai, Songkhla 90110, Thailand

**Keywords:** shellfish, environmental contaminants, *Vibrio*, seasonal dynamics, health risk assessment

## Abstract

Shellfish are an essential component of the human diet, yet their safety is increasingly compromised by contamination with heavy metals, petroleum hydrocarbons, and pathogenic microorganisms, such as *Vibrio*, which pose significant health risks. This study examined shellfish samples from seafood markets, assessing the levels of heavy metals (e.g., cadmium, copper) and petroleum hydrocarbons, while isolating and identifying *Vibrio* species carried by the shellfish. The antimicrobial resistance profiles, resistance genes, and biofilm-forming capacities of these strains were further characterized. Results revealed significant seasonal fluctuations in heavy metal concentrations, with some samples exceeding regulatory limits, indicating potential health risks for long-term consumers. Likewise, *Vibrio* abundance and resistance varied seasonally, with a notable prevalence of multidrug-resistant strains, likely influenced by antibiotic misuse and environmental pressures in coastal regions. Correlation analyses suggested potential links between heavy metal contamination and *Vibrio* resistance, as well as biofilm formation, supporting the hypothesis that metal-induced stress may facilitate resistance gene transfer and enhance biofilm-mediated resistance. This study reveals the seasonal dynamics of antimicrobial resistance (AMR) in shellfish-derived *Vibrio* species and elucidates the dose–response effects of heavy metals and petroleum hydrocarbons, as well as their synergistic selection mechanisms. These findings provide a scientific foundation for assessing shellfish safety, deciphering AMR transmission, and developing ecosystem-based strategies for aquaculture monitoring.

## 1. Introduction

Antibiotic resistance represents one of the most critical threats to global public health security. The ongoing abuse of antibiotics, coupled with the diversity of microbial resistance mechanisms and the horizontal gene transfer of resistance determinants, continues to exacerbate this crisis, posing a severe challenge to current antibacterial treatment strategies. According to the data from the World Health Organization (WHO), multidrug-resistant pathogens cause more than 700,000 deaths annually, and projections suggest that without timely actions—such as rational antibiotic use and the development of new therapeutic strategies against resistant bacterial infections—this number could rise to 10 million by 2050 [1,2]. Notably, the dissemination of resistant bacteria follows the “One Health” framework, in which resistant strains and their genes can spread across the environment, animals, and humans through the food chain, direct contact, or environmental pollution. For example, the abuse of antibiotics in aquaculture can lead to seafood harboring resistant bacteria, which may subsequently enter the human food chain and exacerbate clinical treatment challenges [3,4].

Shellfish are increasingly recognized as a sustainable source of dietary protein, fueling the global expansion of shellfish aquaculture [5]. Farming sites are typically situated in sheltered estuaries and coastal zones, where organic matter–rich waters supply abundant food resources and provide optimal habitats for shellfish growth [6]. Yet, as filter-feeding organisms, shellfish exhibit a pronounced ability to bioaccumulate environmental contaminants, concentrating heavy metals, organic pollutants, and pathogenic microorganisms from surrounding waters [7]. Among these, *Vibrio* spp.—Gram-negative pathogens broadly distributed in marine and estuarine ecosystems—pose dual threats: they drive recurrent *vibriosis* outbreaks in aquaculture with severe economic consequences, and they are major agents of human foodborne disease, causing gastroenteritis, wound infections, and septicemia [8]. Antibiotics remain indispensable for controlling bacterial infections and are extensively applied in aquaculture for disease prevention and treatment. Yet, this heavy reliance frequently drives abuse, accelerating the selection and spread of resistant pathogens. Alarmingly, such resistant strains may enter the human food chain or horizontally transfer resistance determinants to human pathogens via mobile genetic elements, including plasmids and transposons, thereby compounding risks to public health. Mounting evidence indicates that pathogenic *vibrios*, notably *Vibrio parahaemolyticus* and *Vibrio cholerae*, are exhibiting escalating resistance to frontline clinical agents such as β-lactams, aminoglycosides, and fluoroquinolones, with multidrug-resistant isolates being detected at increasing frequency [2].

Beyond antibiotic resistance, the ecological risks posed by marine pollutants demand urgent attention. Rapid industrial and agricultural expansion has introduced vast quantities of toxic substances into marine ecosystems, with heavy metals and petroleum hydrocarbons standing out as dominant contaminants owing to their persistence and potent toxicity [9]. Heavy metals such as Pb, Ni, Fe, Cd, Cr, Mn, and Zn are non-degradable and prone to bioaccumulation, enabling their long-term persistence in aquatic environments and biomagnification through food webs, thereby threatening both aquatic biota and human health [10]. They can enter aquatic organisms via ingestion, gill respiration, or dermal absorption, circulate systemically, and impair host physiology—manifesting as oxidative stress, immunosuppression, reduced growth, or even population collapse [11]. Petroleum hydrocarbons, encompassing complex mixtures of aliphatic and aromatic compounds, largely derive from offshore oil extraction, transport spills, and industrial discharge. Global estimates suggest that roughly 4.63 million tons of petroleum enter the oceans each year [12]. Such inputs disrupt marine ecological balance, restructure biological communities, and bioaccumulate within aquatic organisms, ultimately posing direct risks to human health through seafood consumption. Given the environmental mobility, persistence, and toxicity of heavy metals and hydrocarbons, systematic monitoring and risk assessment are critical for safeguarding marine ecosystems and food safety.

With the escalating global crisis of antibiotic resistance, the study of environmental microbial resistance has emerged as a central theme within the “One Health” framework. Current evidence indicates that resistant bacteria are widespread across diverse environments, with aquatic ecosystems strongly impacted by anthropogenic activities—such as coastal aquaculture zones and urban nearshore waters—harboring particularly high abundances of resistance genes [13]. Of special concern is the role of composite pollution arising from human activity, where the co-occurrence of heavy metals and organic contaminants can foster the development of multidrug resistance through co-selection. Heavy metals may induce cross-resistance in bacterial populations, while certain organic pollutants act as inducers of horizontal gene transfer [14]. Despite notable advances in deciphering the mechanisms of environmental resistance dissemination, our understanding of its key drivers—especially the synergistic interactions of multiple stressors in complex environments—remains limited, and this knowledge gap severely hampers the development of effective resistance mitigation strategies. This work shifts the paradigm from single-metal stress to realistic co-exposure scenarios, systematically dissecting the synergistic effects of heavy metals and petroleum hydrocarbons on host-associated *vibrios*. We focus on their combined impact on antibiotic resistance and biofilm formation—two coupled phenotypes that critically enhance bacterial fitness and pathogenicity.

To address this issue, the present study adopted a monthly sampling strategy to systematically collect shellfish samples available for sale. The study initially performed antimicrobial susceptibility testing and resistance gene identification on *Vibrio* strains isolated from the samples. Additionally, inductively coupled plasma mass spectrometry (ICP-MS) was used to quantitatively measure heavy metal (Cd, Cu, etc.) and petroleum hydrocarbon levels. By integrating microbiological, environmental chemical, and molecular biological approaches, the study aims to investigate the following scientific questions: (1) the seasonal variation in antimicrobial resistance of *Vibrio* strains associated with shellfish; (2) the dose–response relationship between environmental pollutants (heavy metals/petroleum hydrocarbons) and resistance; and (3) the co-selection mechanisms under multi-pollutant exposure conditions. This research links environmental contaminant monitoring with the evolution of microbial resistance, providing novel evidence for understanding the evolutionary dynamics of resistance under complex environmental pressures. More importantly, it offers theoretical support for the development of ecosystem-based aquaculture management strategies and integrated pollution control approaches for coastal zones. The findings have important practical implications for ensuring seafood safety and public health.

## 2. Materials and Methods

### 2.1. Collection of Shellfish Samples

In this study, eight representative and economically important shellfish species commonly sold in seafood markets of China, were selected as research subjects: *Mytilus galloprovincialis*, *Monetaria moneta*, *Sinonovacula constricta*, *Ruditapes philippinarum*, *Meretrix meretrix*, *Mactra antiquata*, *Magallana gigas*, and *Mactra quadrangularis*. These species constitute the principal shellfish consumed by local residents, bearing both economic significance and implications for food safety.

Systematic sampling was conducted from December 2023 to November 2024, thereby covering a complete annual cycle to assess seasonal variation. Each month, 15 samples were collected from seafood markets, yielding a total of 180 valid specimens. Immediately after collection, each specimen was individually sealed in sterile bags, labeled with metadata including sampling date, location, and species, and transported under refrigerated conditions (4 °C) in dedicated cold-chain containers to the lab within 2 h. This protocol ensured both sample freshness and stability of the microbial community. Upon arrival, samples were promptly subjected to standardized preprocessing, including surface sterilization, dissection of soft tissues, and homogenization, thereby generating uniform material for subsequent analyses. The detailed procedure is described as follows:

A rigorous pretreatment protocol was implemented immediately upon sample receipt to safeguard analytical accuracy by removing surface-derived microbial contamination:

Surface Decontamination: This involved an initial scrub with sterile PBS, a disinfecting rinse with 75% ethanol, and a final triple rinse with sterile PBS to ensure no ethanol carryover, followed by drying with sterile filter paper.

Aseptic Dissection: Conducted within a biosafety cabinet, this step utilized autoclaved tools to open the shell and meticulously extract all soft tissues, which were then collected in a pre-weighed, sterile bag.

Homogenate Preparation: After precise weighing, tissues were homogenized in a 1:3 (*w*/*v*) ratio with sterile PBS using a high-throughput homogenizer (Sakezi ×48) (Anhui Shangkezhi Instrument Co., Ltd., Anhui, China) at 60 Hz for 120 s. The resultant homogenate constituted the primary stock for all subsequent culturing analyses.

### 2.2. Isolation and Identification of Vibrio

Approximately 2 g of shellfish tissue was homogenized in 150 μL of phosphate-buffered saline (PBS; Sangon Biotech, Shanghai, China) using a high-throughput tissue grinder (Sakezi × 48) at 60 Hz for 120 s. The homogenates were serially diluted to 10^−3^ fold and spread onto thiosulfate–citrate–bile salts–sucrose (TCBS) agar plates (Qindao Hope Bio-Technology Co., Ltd., Qingdao, China). Following gradient dilution (1:1000) and plating of the homogenate on TCBS selective agar, triplicate cultures per sample were incubated at 28 °C for 12 h, after which yellow-green colonies were randomly selected for isolation, cultivation, and species identification to prevent morphological bias and ensure a representative assessment of *Vibrio* diversity [15]. From each plate, 5–8 representative colonies with typical morphology were randomly selected and inoculated into LB broth (HuanKai Microbial, Guangzhou, China), followed by incubation at 28 °C with shaking for 10–12 h. Genomic DNA was extracted and the 16S rDNA gene was amplified by PCR. The reaction mixture consisted of 12.5 μL of 2× ProTaq Master Mix (AG Bio, Changsha, China), 1 μL each of primers 27F and 1492R (Sangon Biotech, Shanghai, China), and 8.5 μL of ddH_2_O. Amplifications were performed in an automated thermal cycler (Bio-Rad MY_100). PCR products were sequenced commercially (Sangon Biotech, Shanghai, China), and resulting sequences were compared against the NCBI database for species identification. Purified isolates were preserved by mixing with 50% glycerol (Sangon Biotech, Shanghai, China) at a 1:1 ratio and stored at −80 °C in an ultra-low temperature freezer for long-term use.

### 2.3. Antimicrobial Susceptibility Testing and Resistance Gene Detection of Vibrio

Antimicrobial susceptibility was assessed using the Kirby–Bauer (K–B) disk diffusion method against 20 antibiotics: tetracycline (TET), gentamicin (GEN), amikacin (AMK), piperacillin (PIP), ampicillin (AMP), ceftazidime (CAZ), cefazolin (CZ), ceftriaxone (CTR), cefuroxime (CXM), cefoperazone (CPZ), penicillin (PEN), polymyxin B (PB), cephalothin (CN), erythromycin (E), mycinomycin (MY), vancomycin (VAN), kanamycin (KAN), streptomycin (S), minocycline (MI), and doxycycline (DO). Frozen isolates were revived in LB broth, and bacterial suspensions were adjusted to 0.5 McFarland standard (≈1.5 × 10^8^ CFU/mL). Sterile cotton swabs were used to evenly spread the suspensions onto Mueller–Hinton (MH) agar plates (Hopebio HB6232, Qingdao, China). Antibiotic disks (BKMAMLAB) were applied to the agar surface, and plates were incubated at 28 °C for 24 h. Zones of inhibition were measured using a multiparameter imaging analyzer (Shineso Sup G1) (Hangzhou Xunshu Technology Co., Ltd., Hangzhou, China), and susceptibility was interpreted as susceptible (S), intermediate (I), or resistant (R) according to the Clinical and Laboratory Standards Institute (CLSI) guidelines [16].

PCR assays were further employed to detect eight common resistance genes in the isolated *Vibrio* strains, including β-lactamase genes (*bla_PER-1_*, *bla_TEM-1_*, *bla_CMY-2_*, *bla_NDM-1_*), aminoglycoside resistance genes (*strA*, *strB*), and macrolide resistance genes (*ermA*, *ermB*). Primer sequences and expected amplicon sizes are listed in Table S1. Each 25 μL PCR reaction contained 12.5 μL 2× Taq PCR Master Mix (AG Bio, China), 1 μL forward primer (10 μM), 1 μL reverse primer (10 μM), 2 μL template DNA (≈50 ng/μL), and 8.5 μL sterile ddH_2_O. Amplification was performed in a thermal cycler (Bio-Rad MY_100). Amplicons were separated by electrophoresis on 1.5% agarose gels (120 V, 30 min) and visualized using a gel imaging system (BIO-OI OI 1000) (Guangzhou Guangyi Biotechnology Co., Ltd., Guangzhou, China). Known reference strains carrying the respective resistance genes were used as positive controls, and sterile ddH_2_O served as the negative control. All PCR assays were conducted in triplicate to ensure reproducibility [17,18,19,20].

### 2.4. Biofilm Formation Assay

Bacterial suspensions were adjusted to an optical density at 600 nm (OD_600_) of 0.2. Then, 100 µL of each suspension was aliquoted into a 96-well polystyrene microtiter plate, with six biological replicates per strain. The plate was incubated statically at 28 °C for 24 h. After incubation, the planktonic cells were carefully aspirated, and the adhered biofilms were gently washed twice with sterile phosphate-buffered saline (PBS) and air-dried. The biofilms were then stained with 0.1% crystal violet for 15 min at room temperature. Excess stain was removed by washing three times with sterile PBS, and the plate was air-dried completely. The stained biofilm was dissolved using 33% acetic acid, and the absorbance was measured at 590 nm using a multimodal microplate reader (BioTek Synergy H1) (Boten Instrument Co., Ltd., VT, USA) to quantify biofilm formation [21].

### 2.5. Detection of Heavy Metals (Cadmium and Copper) and Petroleum Hydrocarbons in Mollusks

The concentrations of cadmium, copper, and petroleum hydrocarbons in mollusks were determined using flame atomic absorption spectrophotometry and fluorescence spectrophotometry, respectively, as outlined in the National Standard of the People’s Republic of China (GB 17378.6-2007). The detailed methodologies are described as follows.

#### 2.5.1. Determination of Copper

Dried bivalve tissues were digested with HNO_3_–H_2_O_2_ and analyzed by flame atomic absorption spectrometry (FAAS) at 324.7 nm.

(1) Calibration: Standard curves were constructed using copper solutions.
(2) Sample preparation: Approximately 0.2 g of dried tissue was digested and diluted to a fixed volume.
(3) Calculation: Copper content (10^−6^, dry weight) was calculated as follows:

(1) $$\omega_{cu}=\frac{\rho_{cu}V}{M}$$

where ρ_Cu_ is the concentration obtained from the calibration curve (μg/mL), V is the final volume (mL), and M is the sample mass (g).

#### 2.5.2. Determination of Cadmium

Samples were digested with HNO_3_–HClO_4_ and quantified by FAAS at 228.8 nm.

(1) Calibration: Standard curves were constructed using cadmium solutions.
(2) Sample preparation: Approximately 2 g of dried tissue was digested and diluted to 25 mL.
(3) Calculation: Cadmium content (10^−6^, dry weight) was calculated as follows:

(2) $$\omega_{cd}=\frac{\rho_{cd}V}{M}$$

where ρ_Cd_ is the concentration from the calibration curve (μg/mL), V is the final volume (mL), and M is the sample mass (g).

#### 2.5.3. Determination of Petroleum Hydrocarbons

Samples were subjected to alkaline saponification (NaOH)–dichloromethane extraction, residues were dissolved in petroleum ether, and fluorescence spectrophotometry was performed at 310 nm excitation and 360 nm emission.

(1) Calibration: Standard curves were constructed using petroleum hydrocarbon solutions.
(2) Sample preparation: Two to five grams of tissue were saponified, extracted, and diluted.
(3) Calculation: Hydrocarbon content (10^−6^, dry weight) was calculated as follows:

(3) $$\omega_{oil}=\frac{m\cdot V}{F\cdot M}$$

where m is the concentration obtained from the calibration curve (μg/mL), V is the extract volume (mL), F is the dry-to-wet mass ratio, and M is the sample mass (g).

### 2.6. Health Risk Assessment of Heavy Metals in Shellfish

#### 2.6.1. Estimated Daily Intake of Heavy Metals

In this study, the bioaccessibility-adjusted estimated daily intake (EDI) of heavy metals from shellfish was calculated as follows [22]:

(4) $$EDI=\frac{C_{metal}\times W_{shellfish}}{B_{w}}$$

where C_metal_ represents the concentration of heavy metal in shellfish (in μg/kg wet weight), W_shellfish_ denotes the daily consumption rate of shellfish by adults (28.82 g/day) [23], and B_w_ refers to average adult body weight, which was set at 64.87 kg based on data reported by the China National Center for Physical Fitness Surveillance (https://www.sport.gov.cn/n315/n329/c24335066/content.html, accessed on 6 July 2022).

#### 2.6.2. Health Risk Assessment

The target hazard quotient (THQ) for heavy metal exposure through shellfish consumption was evaluated based on the U.S. EPA Region III Risk-Based Concentration Table (US EPA, 2000) using the following equation [24]:

(5) $${THQ}_{i}=\frac{EFr\times ED\times W_{shellfish}\times C_{i}^{metal}}{RfD\times B_{w}\times ATn}$$

where EFr is the exposure frequency (350 days/year), ED is the exposure duration (70 years, equivalent to the average lifespan), W_shellfish_ is the daily intake of shellfish (g/day), $C_{i}^{metal}$ is the concentration of the individual metal in shellfish (μg/kg wet weight), RfD is the oral reference dose, with values set as follows: Cd = 1 μg kg^−1^ day^−1^ (US EPA, 2011), Cu = 40 μg kg^−1^ day^−1^ (US EPA, 2011), B_w_ is the average body weight (kg), and ATn is the average exposure time for non-carcinogens (ED × 365 days/year).

#### 2.6.3. Total Hazard Index (HI) Assessment

For the cumulative risk assessment of multiple heavy metals in shellfish, the total hazard index (HI) was calculated as the sum of the THQ values for all individual metals [25]:

(6) $$HI=\sum_{i=1}^{n}{THQ}_{i}$$

### 2.7. Statistics

We employed GraphPad Prism 10.0, ArcGIS 10.8.1, and Origin 2024 for data processing, visualization, and multivariate correlation analysis.

## 3. Results

### 3.1. Abundance, Seasonal Variation, and Distribution of Vibrio Species

**Figure 1:**
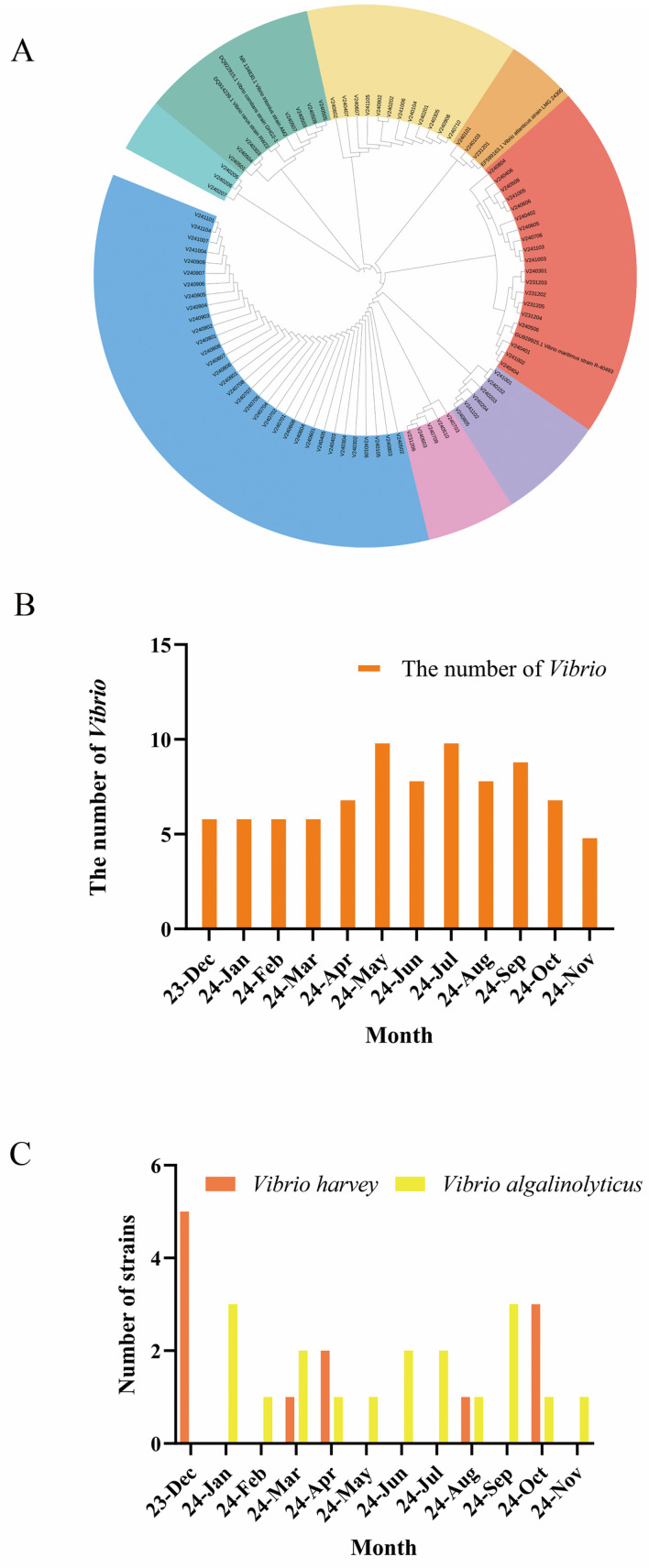
Temporal dynamics and diversity of *Vibrio* populations. (**A**) The phylogenetic analysis of *Vibrio* species isolates derived from shellfish. (**B**) Seasonal distribution of *Vibrio* prevalence in shellfish. (**C**) Monthly dynamics of *Vibrio harveyi* and *Vibrio alginolyticus* isolation.

In this study, a total of 180 shellfish samples were systematically collected from major seafood markets in China. These samples originated from eight key mariculture provinces along the coast of China: Guangdong, Fujian, Guangxi, Jiangsu, Liaoning, Shanghai, Zhejiang, and Shandong. Through selective culture and molecular biological identification, 88 *Vibrio* strains were isolated (Figure 1A). Each strain was designated a unique identifier following the format “V” followed by a number (e.g., V01–V88). Based on 16S rDNA sequence analysis, the isolates were identified as multiple clinically and ecologically significant *Vibrio* species, including *V. alginolyticus*, *V. harveyi*, among others. The species composition and geographical distribution are summarized in Figure 1. A circular phylogenetic tree was constructed using the maximum likelihood method based on 16S rRNA sequences. The root was positioned at the center, with branches radiating outward and terminal leaf nodes (sample labels) arranged along the circumference. Branch lengths correspond to genetic distances, with shorter branches indicating closer phylogenetic relationships and longer branches reflecting higher divergence (as illustrated by cross-color clusters). The tree revealed eight major clades, with samples within each cluster showing high homology, consistent with membership in the same species or subspecies.

Our results indicated significant seasonal fluctuations in *Vibrio* isolation rates (Figure 1B). The highest detection rates were observed in May and July (summer), followed by autumn, while spring and winter yields were comparatively lower. This pattern may be correlated with variations in water temperature and metabolic activity of shellfish. Further analysis of the seasonal distribution patterns of the dominant bacterial species (Figure 1C) revealed that *V*. *harveyi* demonstrated a pronounced winter enrichment, whereas *V*. *alginolyticus* was consistently detected throughout all sampling months.

**Figure 2:**
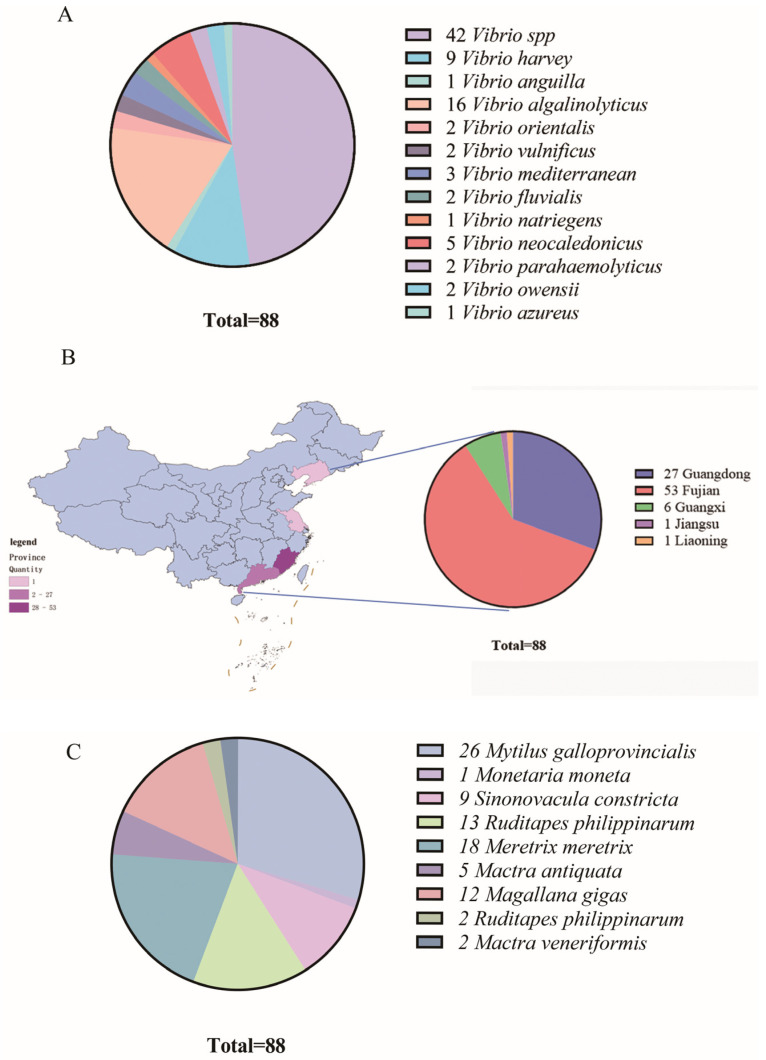
Composition, prevalence, and host distribution of *Vibrio* communities in shellfish. (**A**) Profiling of shellfish-associated *Vibrio* species. (**B**) Prevalence of *Vibrio* spp. in shellfish from different geographical origins. (**C**) Distribution of *Vibrio* spp. across different bivalve hosts.

**Figure 3:**
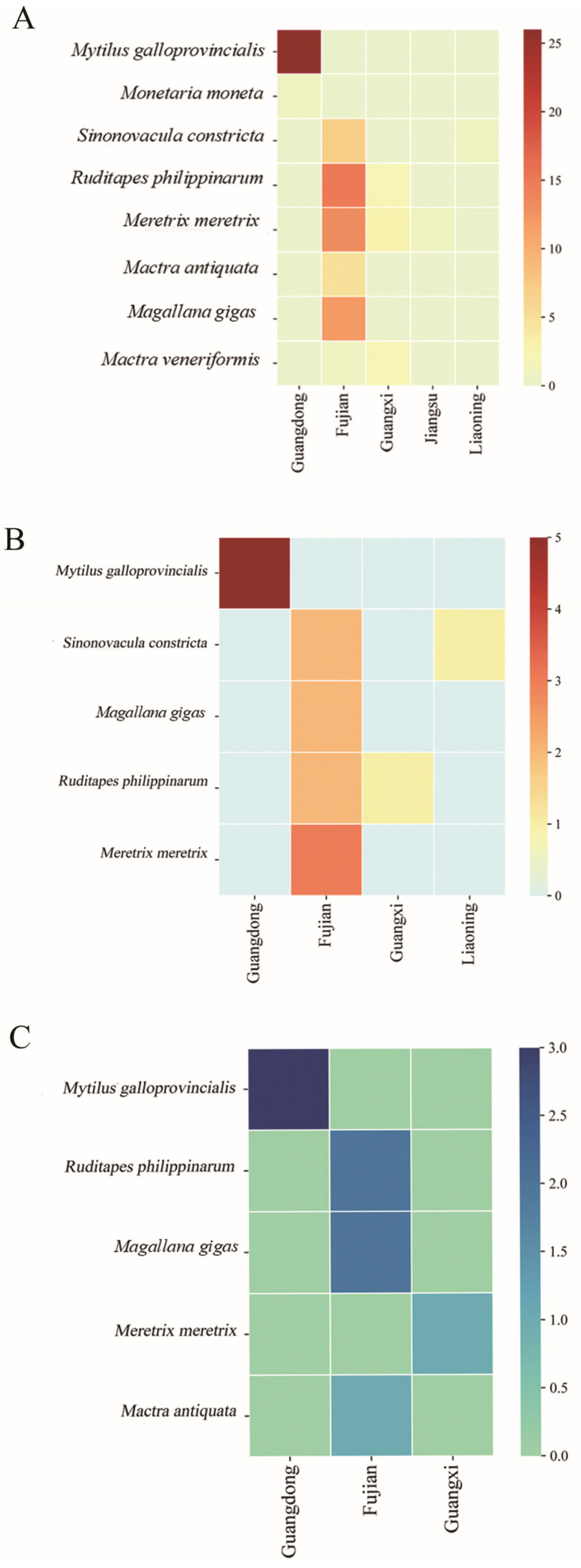
Comparative analysis of host and geographic distribution of *Vibrio* isolates. (**A**) Heatmap analysis of the host and geographic distribution of *Vibrio* spp. isolates. (**B**) Heatmap analysis of the host and geographic distribution of *Vibrio alginolyticus* isolates. (**C**) Heatmap analysis of the host and geographic distribution of *Vibrio harveyi* isolates.

Among the identified *Vibrio* species (Figure 2A), *V. alginolyticus* was the most prevalent, represented by 16 isolates, followed by *V. harveyi* with 9 isolates. Other species, including *V. neocaledonicus*, *V. mediterranei*, and *V. parahaemolyticus*, were also detected. Forty-two strains remained unclassified at the species level. Geographical distribution analysis revealed that the majority of *Vibrio* isolates originated from Fujian Province (53 strains), followed by Guangdong (27 strains). Fewer isolates were obtained from Guangxi (6), Jiangsu (1), and Liaoning (1). No *Vibrio* strains were isolated from samples collected in Shanghai, Shandong, or Zhejiang (Figure 2B). Host-based distribution showed that *Mytilus galloprovincialis* yielded the highest number of *Vibrio* isolates (26 strains), followed by *Meretrix meretrix* (18), *Ruditapes philippinarum* (15), *Magallana gigas* (12), and *Sinonovacula constricta* (9). Lower isolation rates were observed from *Mactra antiquata* (5), *Mactra veneriformis* (2), and *Monetaria moneta* (1) (Figure 2C). Notably, all *M. galloprovincialis* isolates were derived from Guangdong Province. In contrast, isolates from Fujian Province were associated with a wider range of host species, with *R. philippinarum* being the primary host, showing significantly higher colonization rates. *M. meretrix* and *M*. *gigas*. also exhibited substantial *Vibrio* carriage rates (Figure 3A).

Furthermore, distribution analysis of the predominant species, *V. alginolyticus* and *V. harveyi*, across regions and hosts indicated that *V. alginolyticus* was detected in samples from Guangdong, Fujian, Guangxi, and Liaoning. The highest positivity rate was observed in *M. galloprovincialis* from Guangdong. In Fujian, *V. alginolyticus* was also detected in *S. constricta*, *M*. *gigas*, *R. philippinarum*, and *M. meretrix*, with the latter showing particularly high detection rates (Figure 3B). *V. harveyi* was primarily isolated from shellfish in Guangdong, Fujian, and Guangxi, again with the highest rate in *M. galloprovincialis* from Guangdong. Elevated carriage rates were also noted in *R. philippinarum* and *M*. *gigas* from Fujian (Figure 3C).

### 3.2. Antibiotic Resistance and Resistance Gene Detection in Shellfish-Derived Vibrio spp.

**Figure 4:**
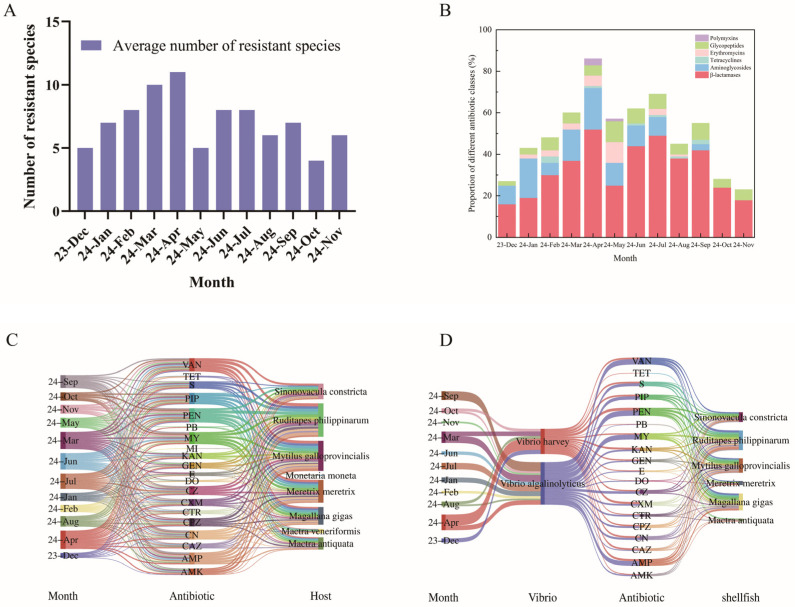
Multidimensional analysis of antimicrobial resistance profiles in shellfish-derived *Vibrio* spp. (**A**) Seasonal dynamics of antibiotic resistance in shellfish-derived *Vibrio* isolates. (**B**) Antimicrobial resistance spectrum of shellfish-associated *Vibrio* populations. (**C**) Seasonal variation in *Vibrio*–antibiotic–host interactions. (**D**) Species-specific seasonal shifts in *V. alginolyticus* and *V. harveyi* interactions with antibiotics and hosts.

The *Vibrio* isolates obtained from shellfish in this study exhibited notable seasonal variations in antibiotic resistance profiles. From December 2023 to April 2024, the number of antibiotic classes to which the isolates showed resistance consistently increased, peaking in April with an average of 12 resistant classes per isolate. A sharp decline to 5 classes was observed in May, followed by fluctuating resistance levels between June and November 2024. Overall, resistance was more prevalent in spring and summer compared to autumn and winter (Figure 4A).

Significant monthly variations were observed in the antibiotic resistance spectra of the *Vibrio* isolates. As shown in Figure 4B, the highest resistance rate was consistently detected against β-lactam antibiotics, followed by resistance to aminoglycosides (except during October–November 2024) and glycopeptides. Notably, a small proportion of isolates resistant to macrolides were detected from January to May and again in July–August.

Table 1 summarizes the antibiotic resistance profiles of shellfish-derived *Vibrio* isolates against 20 antibiotics across different months. All isolates were resistant to penicillin, ampicillin, vancomycin, lincomycin, piperacillin, and cefalexin throughout the study period. Among these, resistance to penicillin (a β-lactam) was the most prevalent (97.73%). Within the aminoglycoside class, 45.45% of isolates were resistant to streptomycin. Resistance to vancomycin (a glycopeptide) reached 76.14%. In contrast, low resistance rates were observed for tetracycline (4.55%), minocycline (2.27%), and polymyxin (4.55%). It is noteworthy that all tested isolates exhibited resistance to at least two antibiotics, with a multidrug resistance rate as high as 97.73%.

Analysis of host species distribution revealed that *M. galloprovincialis* accounted for the highest proportion of resistant isolates, followed by *R. philippinarum*, *M. meretrix*, *M. gigas*, and *S. constricta*. In contrast, lower detection rates of resistant strains were observed in *M. veneriformis* and *M. moneta* (Figure 4C).

Species-specific resistance analysis indicated that *V. alginolyticus* exhibited high resistance to vancomycin (VAN), penicillin (PEN), and ampicillin (AMP), but low resistance to polymyxin B (PB). All *V. alginolyticus* isolates were sensitive to tetracycline (TET). In comparison, *V. harveyi* showed high resistance to PEN, cefuroxime (CXM), and AMP, but low resistance to TET, PB, and doxycycline (DO) (Figure 4D).

**Figure 5:**
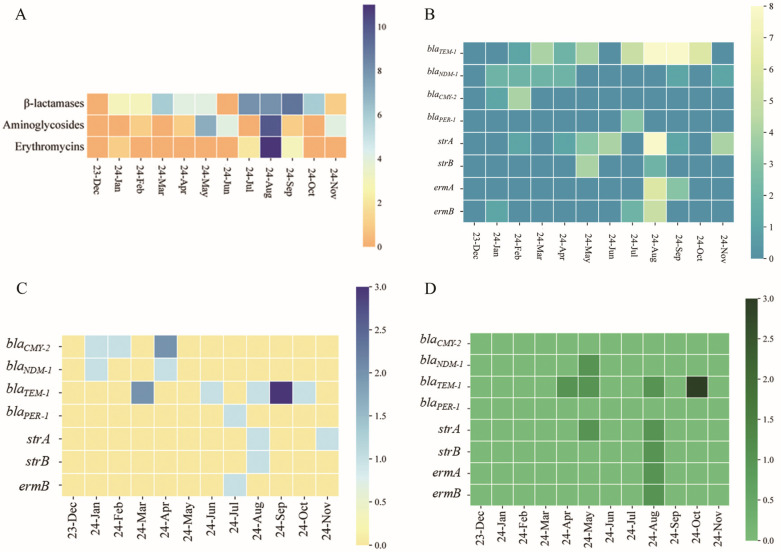
Comparative analysis of the antimicrobial resistance gene profiles in *Vibrio* at the genus and species levels. (**A**) Heatmap of antimicrobial resistance gene prevalence in *Vibrio* spp. by antibiotic class. (**B**) Heatmap of the prevalence of diverse antimicrobial resistance genes in *Vibrio* isolates. (**C**) Antimicrobial resistance gene profile in *Vibrio alginolyticus* isolates. (**D**) Distribution of antimicrobial resistance genes in *Vibrio harveyi* isolates.

Detection of antibiotic resistance genes (ARGs) revealed that β-lactamase genes (including *bla_PER-1_*, *bla_TEM-1_*, *bla_CMY-2_*, and *bla_NDM-1_*) were most frequently detected between July and September, with *bla_TEM-1_* being the most prevalent in August and September. Aminoglycoside resistance genes *strA* and *strB* were more common in May and August, with *strA* peaking in August and *strB* primarily detected during these two months. Macrolide resistance genes were most abundant in August (11 detections). The genes *ermA* and *ermB* were also predominantly detected between July and September, with the highest number of *ermB* occurrences in August (5 detections) (Figure 5A,B).

In *V. alginolyticus*, *bla_TEM-1_* was the dominant ARG in September, while the greatest diversity of ARGs (*bla_TEM-1_*, *strA*, *strB*) was observed in August. For *V. harveyi*, ARGs were most frequently detected in May, August, and October. The highest occurrence of *bla_TEM-1_* was recorded in October, and the broadest spectrum of ARGs was observed in August (Figure 5C,D).

### 3.3. Biofilm Formation Capacity of Vibrio spp.

**Figure 6:**
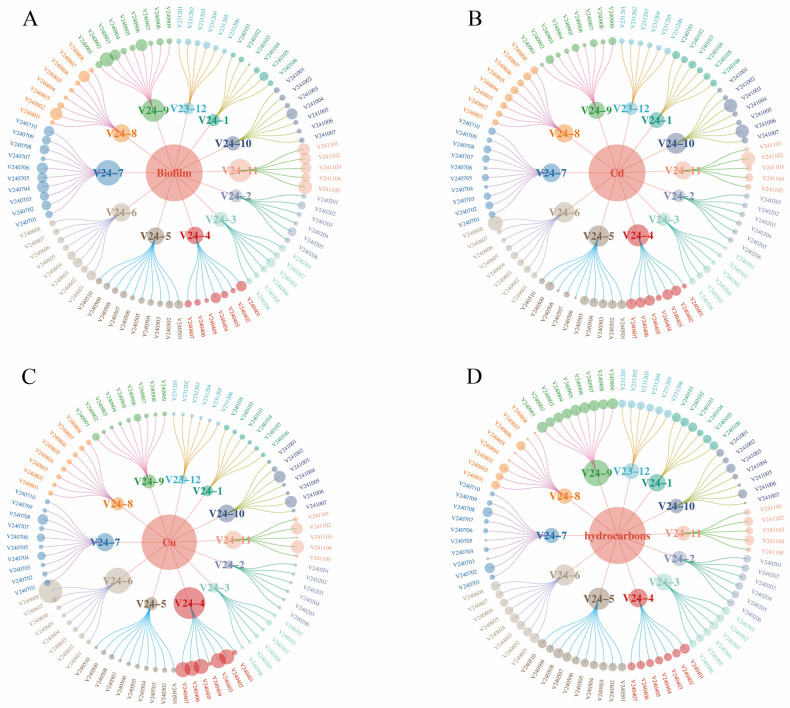
Analysis of biofilm formation in *Vibrio* isolates and heavy metal content in shellfish. (**A**) Assessment of biofilm formation capacity in *Vibrio* isolates. (**B**) Cadmium (Cd) concentration in shellfish tissue. (**C**) Copper (Cu) concentration in shellfish tissue. (**D**) Hydrocarbon concentration in shellfish tissue.

Beyond antibiotic resistance genes, the capacity for biofilm formation represents a key factor contributing to antimicrobial resistance in *Vibrio* spp. As shown in Figure 6A, significant temporal variation in biofilm formation was observed among *Vibrio* strains isolated in 2024. Isolates from July, September, and November predominantly exhibited strong biofilm-forming capacity, whereas those from February showed generally weak activity. Several *Vibrio* strains isolated from shellfish collected in southeastern coastal regions of China (Guangdong, Fujian, Guangxi, and Jiangsu provinces) demonstrated high biofilm-forming ability. Notable biofilm-forming strains included *Vibrio* spp. V240407, V240902, and V241105; *V. parahaemolyticus* V240704 and V241102; *V. neocaledonicus* V240903 and V241101; and *V. alginolyticus* V240906 and V241105. These isolates were obtained from hosts including *R. philippinarum*, *M. gigas*, *S. constricta*, and *M. galloprovincialis*. A clear host-specific pattern was observed, with *R. philippinarum* being the predominant host for high-biofilm formers, followed by *M. gigas* and *S. constricta*.

### 3.4. Cadmium, Copper, and Petroleum Hydrocarbon Content in Shellfish

Statistical analysis of cadmium (Figure 6B), copper (Figure 6C), and petroleum hydrocarbon (Figure 6D) levels in shellfish samples across different months and regions revealed significant spatiotemporal variations.

Elevated cadmium and copper concentrations were detected in April 2024 in several shellfish samples (hosts of strains V240402, V240403, V240405, V240406, and V240407). Notable accumulation was also observed in June (hosts of V240602 and V240603), October (host of V241002), and November (host of V241102). The most affected species were *M*. *gigas*, with some from *M. galloprovincialis*; *M*. *gigas* were the predominant contaminated hosts. Geographically, samples with high heavy metal levels primarily originated from Fujian and Guangdong provinces, especially Fujian. In contrast, petroleum hydrocarbon levels peaked in September 2024, mainly in *R. philippinarum* and *M*. *gigas*, with the former being the most affected. These samples also largely originated from Fujian Province. Substantial intra-species and spatial variability in heavy metal and petroleum hydrocarbon content was observed even within the same month (e.g., May, June, October, and November 2024).

### 3.5. Estimation of Daily Heavy Metal Intake

The Estimated Daily Intake (EDI) was used to quantify human exposure to heavy metals through shellfish consumption and assess associated health risks. Based on measured cadmium and copper concentrations (Table 2), EDI values were calculated. The EDI for cadmium ranged from 0.018 μg kg^−1^ day^−1^ (at the minimum detected level) to 0.849 μg kg^−1^ day^−1^ (at the maximum level), with an average of 0.094 μg kg^−1^ day^−1^. For copper, the EDI values were 0.382, 26.568, and 3.349 μg kg^−1^ day^−1^ at the minimum, maximum, and average levels, respectively. Thus, the estimated daily intake of cadmium through shellfish consumption ranged from 13.36 μg to 55.07 μg, and that of copper from 24.78 μg to 1723.47 μg, based on an average body weight of 64.87 kg.

### 3.6. Risk Assessment of Heavy Metals in Shellfish

The Target Hazard Quotient (THQ) was employed to evaluate non-carcinogenic health risks associated with individual heavy metals through dietary exposure. This metric compares the estimated daily intake with the reference dose (RfD) to determine the acceptability of risk. The Hazard Index (HI), defined as the sum of THQs for all heavy metals studied, was used to assess the cumulative risk posed by simultaneous exposure to multiple contaminants, reflecting their combined toxic effects. THQ values were calculated based on the minimum, average, and maximum concentrations of heavy metals detected in shellfish (Table 3). For cadmium (Cd) and copper (Cu), a THQ value below 1 indicates acceptable risk with no significant health concerns, whereas a THQ ≥ 1 suggests potential health risks requiring intervention. The HI was derived as the sum of the THQs for Cd and Cu. An HI < 1 indicates low cumulative risk, while an HI ≥ 1 signifies an unacceptable level of combined risk, necessitating prioritized management measures. The calculated THQ values for Cd were as follows: THQ_min_ = 0.057, THQ_max_ = 2.712, THQ_ave_ = 0.301. For Cu, the values were: THQ_min_ = 0.009, THQ_max_ = 0.637, THQ_ave_ = 0.053. Thus, across all shellfish samples, the THQ ranged from 0.057 to 2.712 for Cd and from 0.009 to 0.053 for Cu. The resulting HI values ranged from 0.066 to 3.349.

### 3.7. Correlation Analysis of Vibrio Antibiotic Resistance, Biofilm Formation, and Heavy Metal and Petroleum Hydrocarbon Contamination

**Figure 7:**
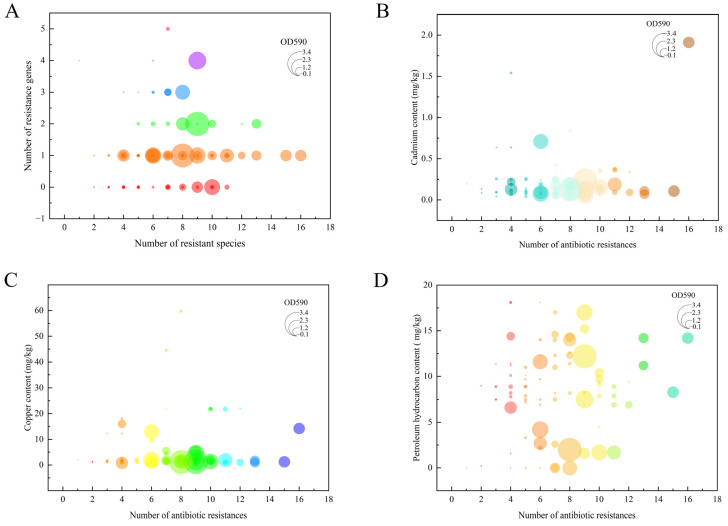
Correlative interplay between antimicrobial resistance, biofilm formation, and environmental stressors. (**A**) Correlation analysis of the number of antimicrobial resistance categories, resistance genes, and biofilm formation capacity. (**B**) Associations among antimicrobial resistance categories, biofilm formation, and cadmium (Cd) contamination levels. (**C**) Interplay between antimicrobial resistance categories, biofilm formation, and copper (Cu) contamination levels. (**D**) Relationships of antimicrobial resistance categories and biofilm formation with total petroleum hydrocarbon (TPH) levels.

Integrated analysis of resistance phenotypes, antibiotic resistance genes (ARGs), and biofilm formation capacity revealed a significant positive correlation between biofilm formation and multidrug resistance (Figure 7A). Specifically, isolates with stronger biofilm-forming ability generally exhibited broader antibiotic resistance profiles. Although the number of ARGs carried by a strain was generally proportional to its resistance spectrum, several exceptions were noted. For instance, *Vibrio orientalis* strain V240603—carrying only one ARG (*bla_TEM-1_*)—exhibited resistance to 10 antibiotics (including CN, AMP, PIP, and AMK). In contrast, a *Vibrio* spp. strain (V240804) carrying three ARGs (*bla_TEM-1_*, *strA*, and *ermB*) was resistant to only one antibiotic (PEN).

Heavy metal levels in shellfish showed certain correlations with both the multiplicity of antibiotic resistance and biofilm formation in *Vibrio* isolates (Figure 7B,C). Increased cadmium and copper concentrations were associated with enhanced multidrug resistance, with a particularly significant positive correlation between Cd levels and the breadth of the resistance spectrum. In contrast, heavy metal contamination appeared to inhibit biofilm formation, as isolates from high Cd/Cu environments generally exhibited reduced biofilm production. Notably, no statistically significant correlation was observed between petroleum hydrocarbon contamination and either antibiotic resistance or biofilm formation capacity (Figure 7D).

**Figure 8:**
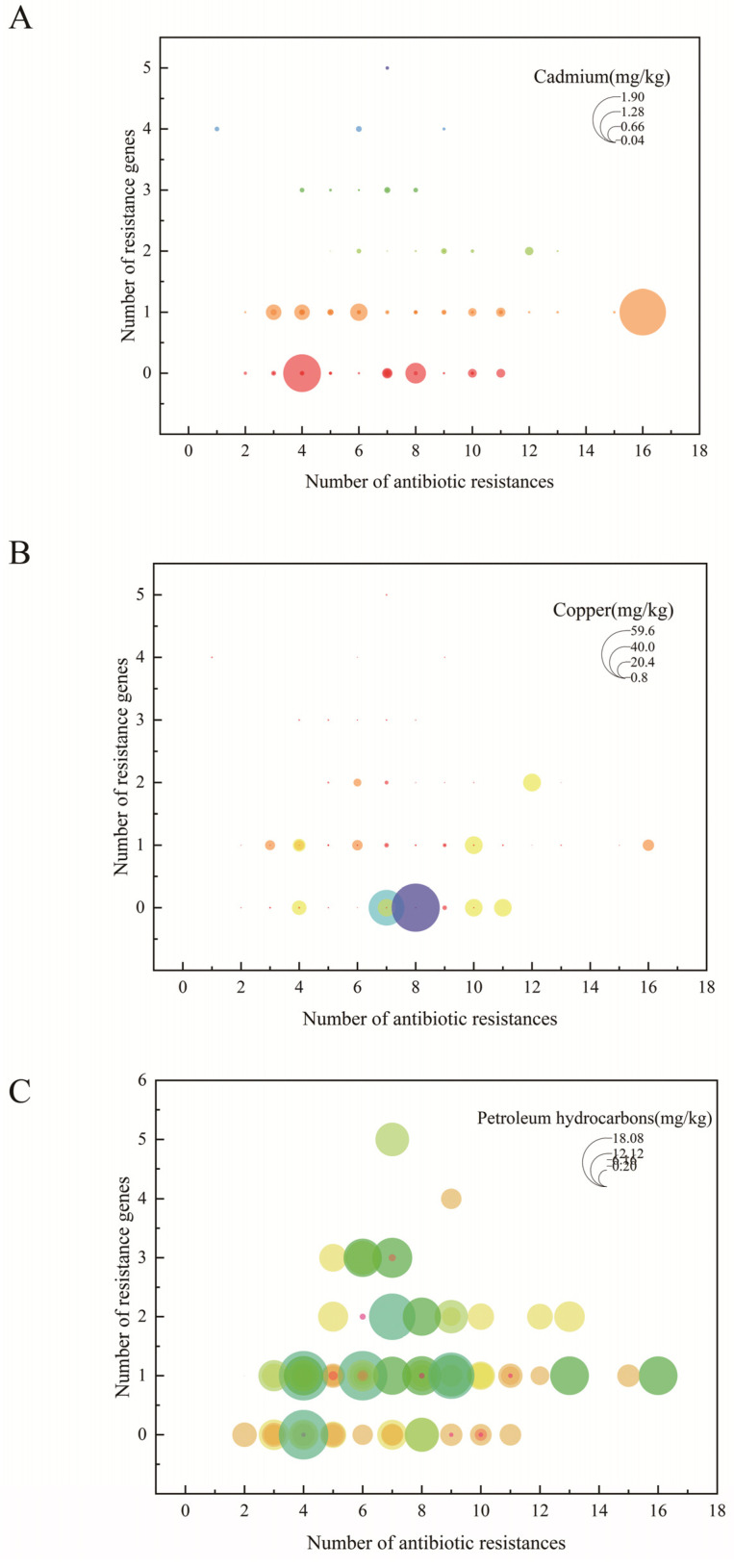
Correlations between antimicrobial resistance traits and environmental contaminants. (**A**) Linking antimicrobial resistance categories, gene abundance, and cadmium (Cd) contamination. (**B**) Associations among AMR phenotypes, resistance genes, and copper (Cu) levels. (**C**) Interplay of antimicrobial resistance traits with total petroleum hydrocarbon (TPH) contamination.

Integrative analysis of phenotypic resistance, ARGs, and environmental contaminants (Figure 8) indicated that *Vibrio* strains carrying multiple ARGs were predominantly isolated from shellfish with lower heavy metal (Cd/Cu) content. Conversely, in environments with substantial heavy metal pollution (particularly Cd and Cu), even strains carrying fewer ARGs displayed broad-spectrum resistance. No clear association was observed between petroleum hydrocarbon levels and either resistance phenotypes or ARG distribution. Collectively, these findings suggest that antibiotic resistance in shellfish-derived *Vibrio* is governed by a multifactorial mechanism. In addition to intrinsic resistance genes, cooperative effects between biofilm formation capacity and environmental contaminants—especially heavy metals—significantly influence resistance phenotypes.

## 4. Discussion

In this study, a total of 88 *Vibrio* strains were isolated from shellfish samples collected across different seasons from selected areas along the southeastern coast of China. The abundance of *Vibrio* isolates exhibited noticeable seasonal fluctuations, as well as geographic and host-specific variations. The prevalence of *Vibrio* in shellfish showed an increasing trend from December 2023 to November 2024, with the highest detection rates observed during the warmer summer months, particularly from May to August. Among all identified *Vibrio species*, *V. alginolyticus* and *V. harveyi* constituted a substantial proportion of the isolates. Notably, *V. alginolyticus* was detected consistently throughout the sampling period, with the exception of December 2023, indicating its persistent presence in the studied shellfish populations. These findings align with previous reports by Böer et al. [26] in the North Sea of Germany.

It is well established that environmental parameters such as temperature and salinity significantly influence the density and diversity of *Vibrio* species [27]. Consistent with our observations, studies such as those by Abioye et al. [28] have demonstrated that temperature is a key driver of *Vibrio* isolation frequency. Numerous investigations across various regions have reported a strong correlation between water temperature and the abundance of *Vibrio* spp. in seawater and shellfish, as well as the incidence of *vibriosis* [26,27,28]. Furthermore, predictive regression models developed by Böer et al. [26] indicate that elevated temperatures exert a strong positive effect on most *Vibrio* species, suggesting a preference of this genus for warmer waters, periods, and seasons. This thermophilic characteristic provides a plausible explanation for the observed seasonal peak in *Vibrio* prevalence.

*Vibrio* spp. also exhibited distinct patterns in geographical and host distribution. Geographically, the majority of *Vibrio* isolates were obtained from shellfish samples collected in Fujian and Guangdong provinces. A higher abundance of isolates was recovered from species such as *M. galloprovincialis*, *R. philippinarum*, *M. meretrix*, and *M*. *gigas*. In marine environments, bivalve mollusks serve as natural reservoirs for *Vibrionaceae*. *Vibrio* species are commonly present as part of the microbiota of healthy oysters and mussels, which can accumulate these bacteria in their tissues and body fluids, including hemolymph. Fujian and Guangdong, as major shellfish aquaculture regions along the southeastern coast of China, experience a subtropical monsoon climate characterized by high annual temperatures, hot and rainy summers, and frequent typhoon events [29,30]. Heavy rainfall often leads to significant reductions in seawater salinity [31], while extensive high-density aquaculture practices can contribute to water quality deterioration [32]—such as through accumulation of residual feed and benthic hypoxia—which may compromise shellfish immunity. Collectively, these factors, including elevated temperatures, salinity fluctuations, and degraded aquaculture conditions, create a conducive environment for the proliferation and transmission of *Vibrio* spp.

*V. alginolyticus* and *V. harveyi* were the most frequently isolated species in this study, predominantly recovered from *M. galloprovincialis* samples in Fujian and Guangdong provinces. Their distribution, however, varied across other shellfish species: *V. alginolyticus* was more frequently detected in *M. meretrix*, whereas *V. harveyi* was more common in *R. philippinarum* and *M. gigas*. These observations align with previous reports. Maria Emanuela Mancini et al. [33] noted that *M. galloprovincialis* harbored the highest abundance of *Vibrio* spp., suggesting that elevated temperatures may impair the bactericidal capacity of hemolymph, thereby facilitating colonization by *V. alginolyticus*. Similarly, Franciska M. Schets et al. [34] reported that *V. alginolyticus* accounted for 80% of *Vibrio* isolates from *M. galloprovincialis* and *Ostreidae*. Sabrina Hossain et al. [35] also identified *M. galloprovincialis* as a major reservoir of *vibrios*, particularly *V. alginolyticus*.

Collectively, our findings and existing literature indicate that *V. alginolyticus* is not only a dominant *Vibrio* species in aquatic and aquaculture environments but also a frequent opportunistic pathogen and key ecological indicator in shellfish farming [36,37,38]. Furthermore, as filter feeders, oysters (Ostreidae) process large volumes of water to obtain nutrients—more than most other bivalves due to their size [39]—which may lead to accumulation of pathogenic *vibrios*. This is compounded by the diverse molecular mechanisms employed by *Vibrio* spp. to infect oysters [40], underscoring their potential role as significant reservoirs for pathogenic strains. Furthermore, comparative analysis of the seasonal distribution patterns of these two dominant *Vibrio* species revealed that *V. harveyi* exhibited a pronounced winter enrichment, suggesting a specialized cold-adaptation mechanism. In contrast, *V. alginolyticus* demonstrated greater environmental adaptability, as evidenced by its persistent detection throughout all sampling months. This disparity indicates that species-specific niche adaptation strategies underlie their distinct seasonal distribution patterns.

Our analysis revealed significant monthly variations in the number of antibiotic classes to which *Vibrio* spp. were resistant, with higher multi-drug resistance rates observed during summer months. A parallel trend was identified across different antibiotic classes, indicating clear seasonal fluctuations in resistance patterns. These variations correlate with seasonal changes in *Vibrio* abundance, suggesting that—in addition to bacterial load—environmental factors such as temperature, salinity, and precipitation may also influence resistance profiles [2]. For instance, elevated seawater temperatures in summer promote *Vibrio* proliferation, leading to intensified antibiotic usage in aquaculture to control infections. This, in turn, exerts sustained selective pressure that favors the emergence of resistant strains [2]. Furthermore, elevated temperatures have been shown to alter bacterial cell physiology and promote biofilm formation, contributing to the development of antibiotic resistance in *Vibrio* species [41]. In this study, we evaluated resistance to multiple antibiotic classes, including β-lactams, aminoglycosides, tetracyclines, glycopeptides, polymyxin B, and macrolides. The *Vibrio* isolates exhibited high rates of resistance to β-lactam antibiotics. Specifically, high resistance was observed against penicillin (PEN), ampicillin (AMP), and cephalothin (CN), with resistance to PEN reaching 97.73%. These findings are consistent with reports of high prevalence of β-lactam resistance among *Vibrio* isolates in multiple countries [42]. Substantial resistance was also detected against aminoglycosides, particularly streptomycin (S), aligning with findings by Dutta et al. [43]. A significant proportion of isolates (76.14%) were resistant to vancomycin (VAN), a glycopeptide, corroborating reports from South Korea [44]. Limited resistance was noted against polymyxin B and tetracyclines (including TET, DO, and MI), consistent with studies from China [45], South Korea [46], and Malaysia [47]. Multidrug resistance (resistance to ≥3 antibiotic classes) was identified in nearly all isolates (97.73%). *V. alginolyticus* and *V. harveyi* were among the most prevalent species identified and exhibited high rates of resistance to β-lactam and glycopeptide antibiotics. Resistant strains of these species were predominantly isolated from bivalves such as *R. philippinarum* and *M. gigas*. The development of resistance to multiple antibiotic classes in *Vibrio* spp. is driven not only by environmental factors but also by diverse molecular mechanisms. These include reduced membrane permeability due to alterations in the outer membrane and modifications of the lipid barrier, which decrease lipopolysaccharide (LPS) fluidity and impede the penetration of antibiotics such as tetracyclines, aminoglycosides, and chloramphenicol [43]. In addition, efflux pump systems—including RND, MATE, and ABC transporters—utilize ATP or proton motive force to extrude a wide range of antibiotics, such as penicillins, streptomycin, and tetracyclines, from bacterial cells [48]. *Vibrio* spp. also confers antibiotic resistance through enzymatic hydrolysis of core antibiotic structures or chemical modification via group transfer to inactivate antimicrobial agents. Among these mechanisms, the hydrolysis of β-lactams by metallo- or serine-β-lactamases is most prevalent [49]. Additionally, enzymatic modification—such as O-nucleotidylation of aminoglycosides mediated by nucleotidyltransferases (ANT)—represents another key resistance strategy [50]. Resistant *Vibrio* strains were predominantly isolated from bivalves including *M. galloprovincialis* and *R. philippinarum*, species previously shown to harbor high *Vibrio* abundances. Under intensive aquaculture conditions, these shellfish are more susceptible to *Vibrio* infections, prompting frequent antibiotic use. The resulting antibiotic pressure, combined with high bacterial densities, fosters the emergence of resistant strains. Furthermore, horizontal gene transfer via mobile genetic elements facilitates the spread of resistance genes to susceptible strains, amplifying the abundance of resistant *Vibrio* within these populations. In summary, as major reservoirs of *Vibrio*, shellfish under intensive farming face elevated infection risks. Environmental changes—such as fluctuations in temperature and salinity—coupled with chronic antibiotic exposure and diverse bacterial resistance mechanisms, drive the emergence of multidrug-resistant *Vibrio*. While most β-lactams, aminoglycosides, and glycopeptides exhibit limited efficacy against *Vibrio* infections, tetracyclines and the antimicrobial peptide polymyxin B remain relatively effective in both prevention and treatment. To further elucidate these resistance mechanisms, we profiled common resistance genes. β-lactamase genes were most frequently detected, with *bla_TEM-1_* identified in 43.18% of isolates. This plasmid-mediated gene, also found in species such as *E. coli* and *P. aeruginosa*, confers resistance to ampicillin, cephalosporins, and other β-lactam antibiotics [51]. Aminoglycoside resistance genes were also frequently detected among the *Vibrio* isolates. Specifically, *strA* and *strB* were identified in 25% and 6.82% of strains, respectively. These genes encode streptomycin phosphotransferases: *strA* encodes APH(3″)-Ib and *strB* encodes APH(6)-Id, which together confer high-level resistance to streptomycin [52]. In addition, macrolide resistance genes *ermA* and *ermB* were detected in 10.23% and 9.09% of the isolates, respectively. The erythromycin ribosomal methylase (Erm) family includes extensively disseminated genes that are often co-located with other resistance determinants on plasmids or transposons (both conjugative and non-conjugative), playing a significant role in resistance to macrolide antibiotics [53]. Furthermore, our study revealed temporal variations in the detection of antibiotic resistance genes (ARGs), both in terms of prevalence and diversity across different months. ARG detection rates were generally higher during the warmer summer months, with August showing the highest prevalence of β-lactamase, aminoglycoside, and macrolide resistance genes compared to other months. This finding is consistent with reports from multiple geographical regions [54]. ARG distribution exhibited marked seasonal dynamics, with higher abundance and detection frequency in summer and lower levels in winter. Several studies suggest that temperature is a key driver governing the seasonal variation of ARGs [55]. Significant monthly variations were observed in the types of detected antibiotic resistance genes (ARGs), indicating that prevention and treatment strategies for *Vibrio* infections in shellfish aquaculture should be tailored to seasonal patterns and avoid blanket antibiotic use. For instance: During January to March, aminoglycosides and macrolides may serve as effective alternatives or complements to β-lactams for preventing or treating *Vibrio* outbreaks; From April to June, macrolides alone appear to retain efficacy against prevalent *Vibrio* strains; In the summer months (July to September), particularly August, β-lactams, aminoglycosides, and macrolides showed reduced effectiveness. Tetracyclines may represent a more reliable option during this period. ARG profiling revealed that in *V. alginolyticus*, β-lactamase genes were consistently detected from January to June (with the exception of May), while aminoglycoside and macrolide resistance genes were more frequently identified from July to October. In contrast, *V. harveyi* exhibited lower overall ARG carriage. Resistance genes in this species were predominantly detected in April, May, August, and October, with the highest abundance and diversity observed in August. These temporal and species-specific patterns highlight the need for tailored antibiotic selection or alternative treatment strategies when managing infections caused by *V. alginolyticus* and *V. harveyi* in shellfish.

Furthermore, this study revealed a lack of deterministic correlation between the presence of the ARGs tested and their corresponding phenotypic expression in *Vibrio* isolates. Our investigation focused specifically on resistance to β-lactams, aminoglycosides, and macrolides—antibiotic classes of critical importance in both aquaculture and clinical settings. The corresponding ARGs are thus considered high-priority targets for surveillance in *Vibrio* populations, which motivated their selection as the primary focus of this work. It is also pertinent to address the observed high prevalence of vancomycin resistance, for which no associated resistance genes (such as *van* genes) were screened. This omission was deliberate, as vancomycin resistance in Gram-negative bacteria—particularly in *Vibrio* species—is generally recognized as an intrinsic trait [43,56]. This intrinsic resistance is attributed to the low permeability of the hydrophobic outer membrane, which acts as an effective barrier against antibiotics that primarily target Gram-positive bacteria, such as vancomycin, thereby preventing the drug from reaching its site of action [56]. This mechanism fundamentally differs from the acquired vancomycin resistance mediated by van genes in Gram-positive bacteria such as Enterococcus [57]. Consequently, the present study prioritized the investigation of phenotypically expressed resistance mediated by acquired resistance genes.

Biofilms are structured microbial communities wherein bacterial cells adhere to surfaces and become encased in a self-produced matrix of extracellular polymeric substances (EPS) [58]. These communities demonstrate significantly enhanced resistance compared to planktonic cells, as the EPS matrix acts as a protective barrier that impedes the penetration of antimicrobial compounds and other foreign agents [59]. Consequently, eradicating biofilms using conventional antibiotics presents considerable challenges. The development of antibiotic resistance is driven by both the frequent use of antibiotics and the horizontal transfer of mobile genetic elements, processes that are often facilitated within bacterial biofilms [60]. To evaluate the biofilm-forming capacity of *Vibrio* isolates, we employed a crystal violet staining assay. Biofilms were stained and subsequently solubilized with 33% acetic acid, followed by measurement of the optical density at 590 nm to quantitatively assess biofilm formation. Our results indicated notable strain-dependent differences in biofilm production; however, no clear seasonal variation was observed across isolates collected in different months.

Cadmium and copper represent two major toxic pollutants in marine environments, capable of inducing oxidative stress, DNA damage, apoptosis, and protein denaturation in various organisms [61]. Bivalves such as mussels have long been employed as effective sentinel organisms due to their ability to accumulate contaminants from the surrounding environment or through the food chain. As filter-feeding organisms, mussels can concentrate metals in their gills and other tissues [62]. Our findings reveal considerable monthly variation in cadmium and copper levels detected in shellfish samples. Notably, elevated concentrations of both metals were observed in April, June, July, October, and November compared to other months. TPH, a priority organic pollutant, constitute a complex mixture comprising alkanes, alkenes, aromatic hydrocarbons, heterocyclic compounds, and other constituents with diverse molecular structures. Anthropogenic activities such as oil spills, shipping operations, direct marine disposal, and industrial waste discharge represent major sources of TPH introduction into marine environments [63]. Once released, TPH can disperse in seawater, adsorb to sediments, and accumulate in biota, eventually being ingested by aquatic organisms such as fish and shellfish [64]. To better understand the accumulation of petroleum hydrocarbons in shellfish and assess potential health risks associated with human consumption, we quantified TPH levels in monthly collected samples. Our results revealed that the highest TPH concentrations occurred in September (autumn), followed by relatively high levels in May, June, July, and August (summer). These findings are consistent with those reported by Xue Wang et al. [65], who also observed elevated TPH levels during autumn and summer. This seasonal pattern may be attributed to reduced riverine input of pollutants during dry seasons and intensified aquaculture activities during rainy periods [66,67]. Based on the detected concentrations of cadmium (Cd) and copper (Cu) in shellfish and the average daily shellfish consumption in China (28.82 g/day), the estimated daily intake (EDI) of Cd and Cu was calculated. The reference dose (RfD) for cadmium is 1.0 μg/kg bw/day, corresponding to a provisional tolerable daily intake of 64.87 μg for an average adult. The daily Cd intake from shellfish consumption in this study ranged from 13.36 μg to 55.07 μg, accounting for 20.60% to 84.89% of the tolerable intake. At the mean Cd concentration, the daily Cd intake was 19.53 μg, representing 30.11% of the RfD—a level considerably higher than that reported by Li et al. [68]. These results suggest that cadmium levels in shellfish may pose potential health risks to consumers. The reference dose (RfD) for copper in this study is 40 μg/kg/day, corresponding to a provisional tolerable daily intake of 2594.8 μg for an average adult. The estimated daily intake (EDI) of copper from shellfish consumption ranged from 24.78 μg to 1723.47 μg, representing 0.95% to 66.42% of the tolerable intake. At the mean concentration, daily copper intake was 142.195 μg, accounting for 5.48% of the RfD—a level higher than that reported by Chai et al. [69]. Substantial variability in metal content was observed among shellfish from different regions and seasons, resulting in significant spatial and temporal differences in daily intake levels. These values indicate an increasing trend compared to earlier studies in China, highlighting the need to monitor heavy metal exposure from shellfish—particularly during late spring, summer, and early winter. The Target Hazard Quotient (THQ) is a comprehensive risk index used to evaluate the non-carcinogenic health risks associated with heavy metal exposure through consumption of contaminated shellfish. The Hazard Index (HI), calculated as the sum of individual THQ values, provides an integrated assessment of the cumulative risk posed by multiple heavy metals. In general, an HI value ≤ 1 suggests no significant adverse health effects, whereas an HI > 1 indicates potential negative health implications. Based on the THQ values derived from varying concentrations of cadmium and copper in the sampled shellfish, the HI ranged from 0.066 to 3.349, with a mean value of 0.354. Substantial variability in heavy metal content was observed across different sampling locations and seasons, resulting in divergent risk levels. Notably, shellfish collected during autumn and summer exhibited elevated metal concentrations, warranting heightened vigilance. Continuous monitoring and risk assessment are essential to prevent excessive heavy metal intake and mitigate potential health hazards. The development of antibiotic resistance in *Vibrio* species constitutes a complex process involving dynamic environment–organism interactions. This study elucidates the mechanistic interplay among biofilm formation, ARGs, and environmental contaminants. We demonstrated a significant positive correlation between biofilm-forming capacity and antibiotic resistance, consistent with previous reports [70]. Notably, certain strains exhibiting strong biofilm formation displayed broad-spectrum resistance despite carrying few ARGs, suggesting that—in addition to genetically encoded resistance—biofilms may enhance tolerance through physical barriers or quorum-sensing-mediated mechanisms [71]. Environmental heavy metal pollution exerted concentration-dependent dual effects on *Vibrio* phenotypes. At sub-inhibitory concentrations, cadmium and copper promoted biofilm formation and increased the abundance of ARGs. This aligns with findings by Hennequin et al. [72] on enhanced biofilm formation under sub-inhibitory metal exposure, and supports observations by Sun et al. [73] regarding metal-induced enrichment of ARGs. Conversely, metal concentrations exceeding tolerance thresholds not only suppressed biofilm formation but also reduced ARG abundance, likely through disruption of cellular integrity [74] and alteration of microbial community structure. These results are consistent with reports by Sallami et al. [75] and Navarrete et al. [76], wherein high metal levels impaired bacterial biofilm formation. Such non-linear dose–response relationships explain why strains harboring numerous ARGs in this study were predominantly isolated from low-metal environments. In summary, at sub-inhibitory concentrations, heavy metals can enhance biofilm formation by inducing bacterial cell adhesion or altering the architecture of the EPS matrix [77]. Furthermore, they promote the selection of antibiotic-resistant bacteria [78] through co-selection (co-resistance or cross-resistance) and other potential mechanisms leading to antibiotic resistance [79]. In contrast, when heavy metal concentrations exceed sub-inhibitory levels, they exert inhibitory effects on these processes. Supra-threshold metal concentrations impair biofilm formation and disrupt cellular structural integrity via interactions with bacterial membrane lipids and proteins. This leads to increased membrane permeability, leakage of cellular contents, redox-catalyzed generation of reactive oxygen species (ROS), and disruption of ion homeostasis, DNA integrity, and protein synthesis [80]. Consequently, high concentrations of heavy metals induce bacterial cytotoxicity, compromise cellular function, and ultimately cause cell damage. These alterations disturb the chemical composition, structure, and functionality of microbial communities, resulting in reduced species composition, abundance, and diversity [81]. As a result, the host microorganisms carrying ARGs decline, leading to an overall reduction in ARG abundance.

Notably, a significant synergistic effect on antibiotic resistance was observed when biofilm formation capacity coexisted with heavy metal exposure, resulting in resistance levels substantially higher than those induced by either factor alone. This synergy may stem from the ability of biofilms to provide a protective microenvironment against metal toxicity, while simultaneously, metal stress promotes the evolution of resistance within the biofilm-associated communities. In contrast, petroleum hydrocarbon contamination, within the concentration range tested in this study, did not show a significant correlation with either antibiotic resistance or biofilm formation in *Vibrio* spp., which may be attributed to its distinct mode of action and selective pressure threshold. These findings provide a novel eco-physiological perspective on the emergence and dissemination of antibiotic resistance in *Vibrio* species within aquatic environments.

Previous research has primarily focused on the selective pressure exerted by individual heavy metals on antibiotic resistance in free-living bacteria from water or sediments. However, real-world aquaculture environments are often complex systems co-contaminated with heavy metals and petroleum hydrocarbons. Furthermore, shellfish, which are significant accumulators of pollutants and natural reservoirs for foodborne pathogens like *Vibrio*, may constitute a unique intra-host microenvironment that serves as a hotspot for resistance evolution. Biofilm formation, a key virulence factor in pathogens, is also a crucial element influencing antibiotic resistance.

To address this knowledge gap, our study provides the systematic investigation of the synergistic effects of combined heavy metal and petroleum hydrocarbon pollution on *Vibrio* species derived from shellfish. This approach more accurately mimics environmental conditions and reveals more complex drivers of resistance and biofilm formation than studies involving single pollutants. We shift the research perspective from traditional focus on bacteria in water and sediments to the critical ecological niche of host-associated *vibrios* within shellfish, which is highly relevant to human health. Beyond antibiotic resistance, we simultaneously analyzed the response of biofilm-forming capacity to environmental stress.

We found that co-exposure to heavy metals and petroleum hydrocarbons enables sub-inhibitory levels of cadmium and copper to co-select for antibiotic resistance and concurrently alter biofilm formation in *vibrios*. This phenotypic coupling likely confers a substantial fitness advantage in hostile environments, promoting the horizontal spread of resistance genes and thereby amplifying virulence. Our findings thus redefine the scope of risk posed by environmental pollutants, introducing a new dimension to pathogen evolution.

A key limitation of this study lies in the scope of heavy metals analyzed. While cadmium and copper were thoroughly investigated due to their known prevalence and toxicity, other potentially harmful metals, including lead (Pb), mercury (Hg), and zinc (Zn), were not included in the risk assessment. These metals are common co-contaminants in many environments and possess significant toxicological profiles. Therefore, our findings likely represent a conservative estimate of the total metal-associated risk. Future research should incorporate a broader spectrum of metals to provide a more comprehensive risk characterization.

## 5. Conclusions

*Vibrio* infections represent a major constraint to the sustainable development of intensive shellfish aquaculture. Environmental factors—including temperature, precipitation, and extreme climatic events—drive pronounced seasonal fluctuations in *Vibrio* abundance by modulating their reproductive dynamics and transmission pathways. Although antibiotics have been widely used to prevent and treat vibriosis, their prolonged and unregulated application has intensified selective pressure, leading to the enrichment of resistant strains. It is noteworthy that no deterministic correlation was observed between the presence of antibiotic resistance genes and their phenotypic expression. Regulatory mechanisms involve multiple pathways, such as efflux pump activation, altered membrane permeability, and target site mutations—processes further modulated by environmental stressors. Additionally, contaminants such as heavy metals (e.g., Cu and Cd) in aquaculture environments not only suppress immune function and growth performance in shellfish but also pose potential risks to human health. Sub-inhibitory concentrations of heavy metals can synergize with biofilm formation to enhance antibiotic resistance in *Vibrio*; however, excessively high metal levels may inhibit biofilm formation and reduce the abundance of resistance genes.

In summary, this study reveals that: (1) Antibiotic resistance in shellfish-derived *Vibrio* strains displays distinct seasonal dynamics, with generally elevated resistance levels in summer and autumn compared to spring and winter. These warmer seasons are characterized by an expansion in the spectrum of antibiotic classes to which *Vibrio* isolates exhibit resistance, alongside an increased detection frequency of antibiotic resistance genes. (2) Environmental heavy metals (Cu and Cd) modulate *Vibrio* antibiotic resistance and biofilm formation in a non-linear, concentration-dependent manner. Specifically, subinhibitory concentrations of heavy metals enhance biofilm formation and elevate the abundance of antibiotic resistance genes, thereby promoting resistance. In contrast, higher metal concentrations suppress biofilm development and reduce resistance gene abundance, leading to diminished resistance. (3) Under co-exposure conditions with heavy metals and petroleum hydrocarbons, heavy metals play a dominant role in shaping *Vibrio* resistance profiles. No significant association was observed between petroleum hydrocarbons and either antibiotic resistance or biofilm formation capacity.

The transmission of antibiotic-resistant *Vibrio* and heavy metal contamination through the food chain—particularly via consumption of raw or undercooked contaminated shellfish—poses significant risks to food safety and public health. As filter-feeding organisms, shellfish can accumulate *Vibrio* species and heavy metals from aquatic environments. When consumed without adequate treatment, they serve as direct vectors for transmitting resistant pathogens to humans, potentially resulting in infections that are more difficult to treat. This pathway underscores an urgent need for integrated risk control strategies to ensure the sustainable development of the shellfish aquaculture industry. Key interventions include: (1) Temperature Management: Maintaining an unbroken cold chain during storage and transport helps suppress *Vibrio* proliferation. More critically, thorough cooking—such as heating until shells open and an adequate internal temperature is reached—effectively inactivates *Vibrio*, interrupting this transmission route. (2) Pollution Monitoring and Source Control: Implementing systematic monitoring of heavy metal levels in aquaculture areas is essential. Concurrently, reducing heavy metal discharges into these environments can help curb the emergence and spread of antibiotic-resistant *Vibrio* at the source. (3) Targeted Awareness Campaigns: It is vital to enhance awareness among consumers and industry stakeholders regarding the health risks associated with consuming raw shellfish, especially those sourced from areas susceptible to anthropogenic pollution.

Collectively, these measures form a multifaceted approach to mitigating the public health threats posed by resistant *Vibrio* and environmental co-contaminants in shellfish.

## Figures and Tables

**Table 1:** Distribution of antimicrobial resistance phenotypes in *Vibrio* isolates.

Antibiotic	R	I	S
CN	40	23	25
PEN	86	2	0
AMK	27	30	31
CPZ	30	35	23
MI	2	0	86
TET	4	0	84
CXM	24	23	41
E	18	63	7
GEN	23	39	26
S	40	36	12
KAN	26	44	18
PB	4	47	37
VAN	67	7	13
DO	6	1	81
CTR	13	4	71
CZ	30	15	43
PIP	48	19	21
CAZ	14	10	64
AMP	53	14	21

**Table 2:** Estimated daily intake (EDI) of cadmium (Cd) and copper (Cu).

EDI			
Heavy Metal	Minimum	Average	Maximum
Cadmium	0.018	0.094	0.849
Copper	0.382	2.192	26.568

**Table 3:** Target hazard quotient (THQ) and hazard index (HI) for cadmium (Cd) and copper (Cu).

THQ_i_			
	Minimum	Average	Maximum
Cadmium	0.057	0.301	2.712
Copper	0.009	0.053	0.637
HI	0.066	0.354	3.349

## Data Availability

The original contributions presented in this study are included in the article/Supplementary Material. Further inquiries can be directed to the corresponding authors.

## Supplementary Materials

The following supporting information can be downloaded at https://www.mdpi.com/article/10.3390/microorganisms13112522/s1. Table S1. Primer sequences and PCR parameters for detection of targeted resistance genes. Table S2. Distribution of antimicrobial resistance phenotypes in *Vibrio* isolates. Table S3. Estimated daily intake (EDI) of cadmium (Cd) and copper (Cu). Table S4. Target hazard quotient (THQ) and hazard index (HI) for cadmium (Cd) and copper (Cu).
