# Supplementary material for: Heavy Metal and Petroleum Hydrocarbon Contaminants Promote Resistance and Biofilm Formation in Vibrio Species from Shellfish
Source: Microorganisms. 2025 Nov 2;13(11):2522. doi: 10.3390/microorganisms13112522 (PMC12654622; doi:10.3390/microorganisms13112522)
Supplement: Supplementary file 1 [file microorganisms-13-02522-s001.zip › microorganisms-3893729-supplementary Tables.pdf]

Table S1. Primer sequences and PCR parameters for detection of targeted resistance genes.

Resistance genes	Sequence	length	Annealing temperature	Primer source
bla*PER-1*	F: GCTCCGATAATGAAAGCGT<br>R: TTCGGCTTGACTCGGCTGA	520 bp	56°C	[17]
*bla*TEM-1	F: CATTTCCGTGTGTCGCCTTATTC<br>R: CGTTCATCCATAGTTGCCTGAC	800 bp	56°C	[17]
*bla*CMY-2	F:GCTGAGAGCTCATGATGAAAAAATCG<br>R: GGTACGGATCCTTATTGCAGC	1146 bp	56°C	[17]
*bla*NDM-1	F: GGTTTGGCGATCTGGTTC<br>R: CGGAATGGCTCATCACGATC	621 bp	55°C	[18]
strA	F: CTTGGTGATAACGGCAATTC<br>R: CCAATCGCAGATAGAAGGC	548 bp	55°C	[19]
strB	F: ATCGTCAAGGGATTGAAACC<br>R: GGATCGTAGAACATATTGGC	509 bp	56°C	[19]
ermA	F: TAACATCAGTACGGATATTG<br>R: AGTCTACACTTGGCTTAGG	139 bp	48°C	[20]
ermB	F: CCGAACACTAGGGTTGCTC<br>R: ATCTGGAACATCTGTGGTATG	200 bp	54°C	[20]

Table S2 . Distribution of antimicrobial resistance phenotypes in *Vibrio* isolates.

Antibiotic	R	I	S
CN	40	23	25
PEN	86	2	0
AMK	27	30	31
CPZ	30	35	23
MI	2	0	86
TET	4	0	84
CXM	24	23	41
E	18	63	7
GEN	23	39	26
S	40	36	12
KAN	26	44	18
PB	4	47	37
VAN	67	7	13
DO	6	1	81
CTR	13	4	71
CZ	30	15	43
PIP	48	19	21
CAZ	14	10	64
AMP	53	14	21

Table S3. Estimated daily intake (EDI) of cadmium (Cd) and copper (Cu)

	EDI		
Heavy metal	Minimum	Average	Maximum
Cadmium	0.018	0.094	0.849
Copper	0.382	2.192	26.568

Table S4. Target hazard quotient (THQ) and hazard index (HI) for cadmium (Cd) and copper (Cu)

	THQ_i		
	Minimum	Average	Maximum
Cadmium	0.057	0.301	2.712
Copper	0.009	0.053	0.637
HI	0.066	0.354	3.349
